# Association between sequence variants in panicle development genes and the number of spikelets per panicle in rice

**DOI:** 10.1186/s12863-017-0591-6

**Published:** 2018-01-15

**Authors:** Su Jang, Yunjoo Lee, Gileung Lee, Jeonghwan Seo, Dongryung Lee, Yoye Yu, Joong Hyoun Chin, Hee-Jong Koh

**Affiliations:** 1 Department of Plant Science, Plant Genomics and Breeding Institute, Research Institute for Agriculture and Life Sciences, Seoul National University, Seoul, 08826 South Korea; 2 Graduate School of Integrated Bioindustry, Sejong University, 209, Neungdong-ro, Gwangjin-gu, Seoul, 05006 South Korea

**Keywords:** Rice, Single nucleotide polymorphisms, Haplotype, The number of spikelets per panicle, Panicle-related traits, Candidate gene association analysis

## Abstract

**Background:**

Balancing panicle-related traits such as panicle length and the numbers of primary and secondary branches per panicle, is key to improving the number of spikelets per panicle in rice. Identifying genetic information contributes to a broader understanding of the roles of gene and provides candidate alleles for use as DNA markers. Discovering relations between panicle-related traits and sequence variants allows opportunity for molecular application in rice breeding to improve the number of spikelets per panicle.

**Results:**

In total, 142 polymorphic sites, which constructed 58 haplotypes, were detected in coding regions of ten panicle development gene and 35 sequence variants in six genes were significantly associated with panicle-related traits. Rice cultivars were clustered according to their sequence variant profiles. One of the four resultant clusters, which contained only *indica* and *tong-il* varieties, exhibited the largest average number of favorable alleles and highest average number of spikelets per panicle, suggesting that the favorable allele combination found in this cluster was beneficial in increasing the number of spikelets per panicle.

**Conclusions:**

Favorable alleles identified in this study can be used to develop functional markers for rice breeding programs. Furthermore, stacking several favorable alleles has the potential to substantially improve the number of spikelets per panicle in rice.

**Electronic supplementary material:**

The online version of this article (10.1186/s12863-017-0591-6) contains supplementary material, which is available to authorized users.

## Background

In rice, the number of spikelets per panicle (SPP) is an important agronomic characteristic that has a strong impact on yield. SPP is determined by several panicle-related traits, such as the number of primary branches per panicle (PB), the number of secondary branches per panicle (SB), and panicle length (PL). Improving the balance between panicle-related traits is needed to optimize SPP [1]. Panicle-related traits are controlled by a range of panicle development genes such as *ABERRANT PANICLE ORGANIZATION 1* (*APO1*) [2], *ABERRANT PANICLE ORGANIZATION 2* (*APO2*) [3], *GRAIN NUMBER 1a* (*GN1a*) [4], *DENSE AND ERECT PANICLE 1* (*DEP1*) [5], *GRAIN NUMBER, PLANT HEIGHT, AND HEADING DATE 8* (*GHD8*) [6], *HEADING DATE 1* (*HD1*) [7], *FLORAL ORGAN NUMBER 1* (*FON1*) [8], *SHORT PANICLE 1* (*SP1*) [9], *LAX PANICLE 1* (*LAX1*) [10], and *MONOCULM 1* (*MOC1*) [11]. A better understanding of the roles and influence of these genes is needed to increase SPP in commercial rice varieties. It is important to identify DNA sequence variation of genes controlling agronomic traits and discover beneficial sequence variants for some traits in that they allow opportunity for molecular application in rice breeding to improve target traits [12].

Candidate gene association analysis investigates the relation between polymorphic sites in genes which are involved in specific phenotypes and phenotypic variations, thereby facilitating identification of causative sequence variants for particular characteristics leading to phenotypic variation. Candidate gene association analysis was previously used to identify novel beneficial polymorphic sites related to important agronomic traits. For example, some functional mutations of *GHD7* that were not identified in an earlier study were detected using General Linear Model (GLM) association analysis [13, 14] and in a separate study, Wei et al. [15] found that five INDELs in the coding region of *HD1* were significantly associated with flowering date in rice.

Functional markers that are directly derived from polymorphic sites within the genes responsible for variations in the target trait can be used directly for marker-assisted breeding as the most effective marker. Prior to identifying the favorable alleles that can be used as resource of functional marker, genetic information about genes, such as level of DNA polymorphism and linkage disequilibrium (LD), are required for successful marker development [16].

The objectives of this study were to (1) identify novel sequence variants in the coding regions of panicle development genes which were previously reported to control panicle development and investigate genetic information, (2) identify sequence variants associated with panicle-related traits, including PL, PB, and SB, and (3) find combinations of favorable alleles for each trait that could contribute to increasing SPP in rice.

## Methods

### Plant materials and phenotypic data analysis

Panicle size of 205 rice varieties were measured and 45 rice varieties were selected using proportionate stratified sampling to ensure that phenotypic variation for panicle size was fully represented (Additional file 1). All plant materials were grown in the experimental field of Seoul National University in Suwon, Korea (37°N latitude). Selected 45 varieties originated from 11 countries and were of three types: *japonica* (*N* = 22), *indica* (*N* = 18), and *tong-il* (*N* = 5) (Additional file 2). Panicle-related traits, including PL, PB, SB, and SPP, were measured using the longest panicle from stem of an individual plant. Three to five replicate measurements were performed in 2013 and 2014. All statistical analysis for phenotypic values was performed using IBM SPSS STATISTICS 21.

### DNA extraction, PCR amplification, and sequencing

Fresh young leaves were harvested from each field-grown plant and stored at −80 °C. Genomic DNA was extracted according to the modified cetyl-trimethyl ammonium bromide (CTAB) method [17]. To identify nucleotide polymorphisms in the coding regions of ten genes associated with panicle development, 48 Primers for sequencing were designed based on Nipponbare reference genome using Primer3plus [18]. Four allele-specific primers for *MOC1* were designed based on sequence data of 8 representative varieties using BatchPrimer3 [19] (Additional file 3). Reactions were performed in a total volume of 50 μl and contained 100 ng genomic DNA, 2.5 nM each primer, 2.5 mM dNTPs, 5 μl 10× buffer, and 0.25 unit Ex Taq (Takara). PCR was performed using a DNA Engine Tetrad 2 Thermal Cycler (Bio-Rad) with the following parameters: 95 °C for 10 min, 30 cycles of amplification (45 s at 95 °C, 45 s at the appropriate Tm for each primer pair, and 72 °C for an appropriate time for product length), and final extension at 72 °C for 10 min. PCR amplicons were separated by gel electrophoresis on 1.5% agarose gels containing ethidium bromide in 0.5× TBE buffer, and visualized using a CDU-2126 Dual UV Trans illuminator (Core Bio System). PCR products were sequenced after purification using a Gel & PCR Purification kit (Inclone Biotech). Sanger sequencing was performed using the BigDye Terminator v3.1 Cycle Sequencing kit with the subsequent analysis on an ABI3730XL automated DNA analyzer (Applied Biosystems).

### DNA sequence analysis

Coding and non-coding regions of 10 genes, including *APO1*, *APO2*, *GN1a*, *DEP1*, *GHD8*, *HD1*, *FON1*, *SP1*, *LAX1*, and *MOC1* were identified by comparison with annotated DNA sequences from the Rice Annotation Project Database IRGSP-1.0 [20]. Multiple sequence alignment was performed using Clustal W [21] and further edited using Bioedit 7.2.0 [22] (Additional file 4). DnaSP 5.0 [23] was used to analyze nucleotide diversity (*π*). Neutrality tests were conducted for calculating Tajima’s *D* [24] by the same program. Haplotype networks separated by mutational steps, including INDELs, were constructed using TCS 1.21 [25]. Tassel 5.2.15 [26] was used to construct UPGMA (Unweighted Pair Group Method with Arithmetic Mean) trees and calculate LD between pairs of polymorphic sites. Only common alleles were used for LD analysis, and SNPs with minor allele frequency (MAF) less than 0.05 were not included.

### Population structure and candidate gene association analysis

Population structure was estimated from 122 SNPs using STRUCTURE 2.3.4 [27]. Ten replicated runs were performed with the following setup: population number of 2–7, burn-in of 50,000, MCMC replication of 100,000, and model for admixture and correlated allele frequencies. The most probable number for *K* was calculated according to Evanno’s methods [28] using Structure Harvester 0.6.94 [29]. Ten Q-matrices obtained from STRUCTURE were combined and permutated using CLUMPP 1.1.2 [30]. Associations between each of the phenotypic values and DNA polymorphisms with MAF >0.05 were analyzed using GLMs in Tassle 5.2.15.

## Results

### Nucleotide diversity

Eight representative varieties were sequenced to find sequence variant in coding region of *MOC1* gene (Additional file 1). Only one non-synonymous SNP (C-G) located at 131 bp was detected by comparison alignment with the Nipponbare sequence as reference (Additional file 5A) and allele-specific primer sets were developed to assess this SNP. Result of SNP genotyping for *MOC1* was presented in Additional file 5B. The *MOC1-*C allele was detected in 35 varieties and mainly distributed in *japonica* varieties (62.8%), and the *MOC1*-G allele was observed in ten varieties, including eight varieties of *indica* and two *tong-il*. A total length of coding region of *LAX1* is 649 bp and only one SNP (T-G) which caused change of amino acid was detected at 349 bp (Additional file 5C). The *LAX1-*T and *LAX1-*G alleles were detected in 18 and 27 varieties, respectively. Of the varieties carrying the *LAX1-*T allele, 75% were of the *japonica* type, and 72% of the varieties carrying the *LAX1-*G allele were of the *indica* type. For the other eight panicle development genes, values of *π* (nucleotide diversity) ranged from 1.44 × 10^−3^ (*GN1a*) to 6.16 × 10^−3^ (*GHD8*). *π* for *APO1*, *APO2*, *DEP1*, and *GHD8* were higher in *indica* than in *japonica* varieties, whereas *π* for *FON1*, *GN1a*, *HD1*, and *SP1* were higher in *japonica* than in *indica*.

### Haplotype diversity

In total, 58 haplotypes were constructed based on polymorphic sites in coding regions of panicle development genes. *Hd* (haplotype diversity) for eight of the ten genes (all except *MOC1* and *LAX1*) ranged from 0.533 (*APO1*) to 0.812 (*GHD8*) (Table 1) and was higher in *indica* than in *japonica* varieties. Haplotypes for *DEP1*, *GN1a*, *HD1*, and *GHD8* genes were known from previous studies [5, 15, 31, 32], but this study was the first to perform haplotype analysis for *APO1*, *APO2*, *FON1*, and *SP1*.

**Table 1:** Summary of DNA variation in nine genes involved in panicle development

Gene (bp)	Group	No. of nucleotide substitutions	No. of INDELs	*π* × 10^−3^	*h*	*Hd*	*D*
*LAX1* (648)	Total	1	0	0.76	2	0.491	–
	*Indica*	1	0	0.66	2	0.425	–
	*Japonica*	1	0	0.14	2	0.091	–
	*Tong-il*	1	0	0.62	2	0.400	–
*APO1* (1299)	Total	12	3	2.02	3	0.533	−0.146
	*Indica*	12	3	3.6	3	0.667	1.135
	*Japonica*	0	0	–	–	–	–
	*Tong-il*	2	1	0.62	2	0.400	−0.973
*APO2* (1170)	Total	13	0	4.83	3	0.536	2.755**
	*Indica*	13	0	4.33	3	0.602	1.329
	*Japonica*	0	0	–	–	–	–
	*Tong-il*	11	0	3.76	2	0.400	−1.200
*DEP1* (1281)	Total	19	1	2.78	9	0.723	−1.251
	*Indica*	9	0	2.38	7	0.854	0.647
	*Japonica*	11	1	1.49	3	0.267	−1.320
	*Tong-il*	6	0	2.03	3	0.700	−0.668
*FON1* (2985)	Total	14	0	2.05	6	0.764	2.820**
	*Indica*	6	0	0.99	4	0.731	2.321*
	*Japonica*	12	0	2	3	0.552	2.812**
	*Tong-il*	10	0	1.34	2	0.400	−1.193
*GHD8* (903)	Total	16	6	6.16	9	0.812	1.605
	*Indica*	15	6	4.07	7	0.866	−0.560
	*Japonica*	11	1	1.54	3	0.495	−1.920*
	*Tong-il*	11	3	7.13	3	0.800	1.527
*GN1a* (1706)	Total	8	4	1.44	8	0.790	0.948
	*Indica*	5	2	0.98	6	0.801	0.526
	*Japonica*	6	3	1.26	4	0.557	0.895
	*Tong-il*	3	2	1.06	2	0.600	1.573
*HD1* (1353)	Total	12	9	4.04	9	0.771	3.105**
	*Indica*	10	8	3.07	6	0.760	1.503
	*Japonica*	11	4	4.11	4	0.686	3.050***
	*Tong-il*	12	4	4.08	3	0.700	−1.031
*SP1* (1881)	Total	20	3	2.11	7	0.679	−0.436
	*Indica*	7	0	1.33	4	0.696	0.756
	*Japonica*	17	3	2.44	5	0.538	−0.105
	*Tong-il*	3	0	0.65	2	0.400	−1.049

*S*, Number of variable sites; *π*, Nucleotide diversity; *h*, Number of haplotypes; *Hd*, Haplotype diversity; *D*, Tajima’s *D*

*, **, and ***, significant at *p <* 0.05, *p* < 0.01, and *p <* 0.001, respectively

**Fig. 1:**
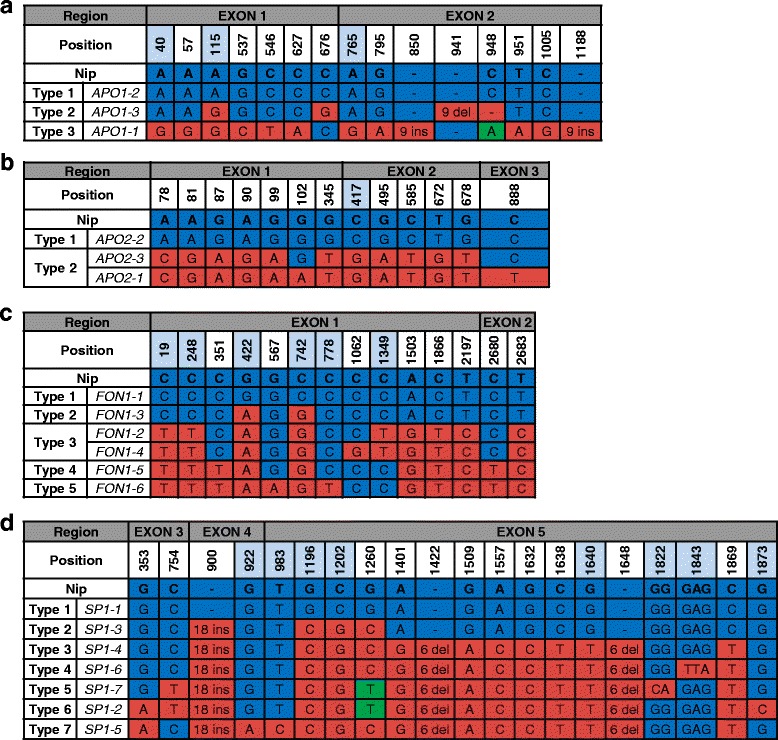
Haplotype analysis of coding sequences. (**a**) *APO1* (**b**) *APO2* (**c**) *DEP1* (**d**) *FON1*. Multiple sequence alignment was performed based on the Nipponbare rice reference sequence. Light blue boxes denote positions of non-synonymous polymorphisms. Nip, Nipponbare; Type, encoded protein type; ins, insertion; del, deletion

**Fig. 2:**
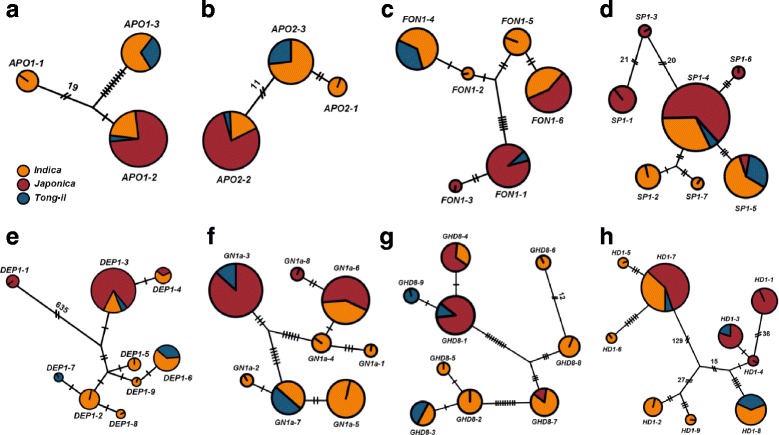
Haplotype networks. (**a**) *APO1* (**b**) *APO2* (**c**) *DEP1* (**d**) *FON1* (**e**) *GHD8* (**f**) *GN1a* (**g**) *HD1* (**h**) *SP1*. Circle size denotes relative haplotype frequency in all cultivars. Colors represent three rice types: *indica*, orange; *japonica*, red; *tong-il*, blue. Dashes and diagonal dashes with numbers between circles denote mutational steps, including gaps in the alignment

Three haplotypes were constructed for the *APO1* coding region, designated *APO1–1* to *APO1–3*, based on 15 polymorphic sites (Fig. 1a). Each haplotypes encoded three different protein types, based on three non-synonymous SNPs and three INDELs. *APO1–2* haplotype was most prevalent, being found in 60% of the 45 rice accessions and all *japonica* varieties carried this haplotype. By contrast, *APO1–1* was found exclusively in four *indica* varieties. *Tong-il* type varieties primarily carried *APO1–3* (Fig. 2a).

Three haplotypes were defined for the *APO2* coding region based on 13 SNPs. *APO2–1* and *APO2–3* encoded identical proteins as the SNPs at 102 and 888 bp position were synonymous SNP (Fig. 1b). *APO2*–*2* was the most frequent haplotype and was carried by 27 varieties, 77.8% of which were *japonica*. *APO2–1* and *APO2–3* were primarily found in *indica* varieties (Fig. 2b). Four *tong-il* type varieties carried *APO2–3*, and one carried *APO2–2*.

Six haplotypes encoding five different protein variants were found for *FON1* (Fig. 1c). The most frequent haplotype was *FON1–6*, which was found in 14 varieties. *FON1–1* and *FON1–4* were detected in 13 and 11 varieties, respectively. Together, these three haplotypes were 84.4% of all accessions. *FON1–2* and *FON1–3* were minor haplotypes, each observed in only one variety (Fig. 2c). Four of the *tong-il* type varieties possessed the *FON1–4* haplotype, and the remaining variety carried *FON1–1*.

Seven haplotypes, each encoding different protein variants, were identified for *SP1*. Different types of protein were predicted by eleven SNPs that led to change of eight amino acid and three INDELs. An 18 bp INDEL was identified specifically in the *SP1–1* haplotype, which was found only in *japonica* varieties (Fig. 1d). Haplotypes *SP1–3* and *SP1–6* were also only found in *japonica*. Conversely, *SP1–2* and *SP1–7* were found exclusively in *indica* varieties. Four *tong-il* type varieties carried the *SP1–5* haplotype. The most prevalent haplotype was *SP1–4*, which was found in 22 varieties. *SP1–3*, *SP1–6,* and *SP1–7* were minor haplotypes, each detected in only one variety (Fig. 2d).

### Phenotypic variations and candidate gene association analysis

Phenotypic values from two field tests are presented in Additional file 6. Pearson correlation coefficients between panicle-related traits (PL, PB, SB, and SPP) were calculated using pairwise correlation analysis. PL showed significant positive correlation with PB, SB, and SPP. Both PB and SB were significantly positively correlated with SPP (Additional file 6). Considering population structure data (Additional file 2, 7 and 8), we conducted GLM association analysis to identify associations between polymorphic sites of ten genes and three panicle-related traits (PL, PB, and SB).

**Fig. 3:**
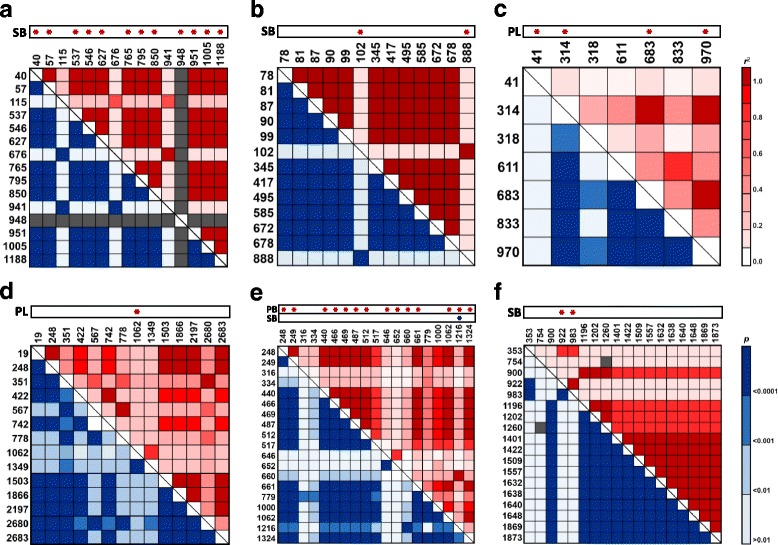
Linkage disequilibrium patterns (MAF <0.05) and polymorphic sites associated with panicle-related traits in two field tests. (**a**) *APO1* (**b**) *APO2* (**c**) *DEP1* (**d**) *FON1* (**e**) *HD1* (**f**) *SP1*. PL, panicle length; PB, the number of primary branches per panicle; and SB, the number of secondary branches per panicle. Asterisks indicate significant associations between sequence variants and phenotypic variations (*p* < 0.05)

Ten SNPs and two INDELs in *APO1* were significantly associated with SB in both field tests (*p < 0.05*). Eleven of the twelve polymorphisms, located at 40, 57, 537, 546, 627, 765, 795, 850, 951, 1005, and 1188 bp, were in complete LD (*r*^*2*^ = 1.0 and *p* = 0.0001; Fig. 3a). The polymorphic site at position 948 bp was detected differentially between the three haplotypes. Polymorphic sites in *APO1* explained 18–19.1% and 11–12.3% of phenotypic variation in the first and second years, respectively (Table 2). Two SNPs in complete LD (102 and 888 bp) in *APO2* were associated with SB in both field tests (Fig. 3b). These SNPs distinguished *APO2–1* from the other haplotypes and explained 12.3% and 11.2% of SB variation in 2013 and 2014, respectively (Table 2). In *DEP1*, four SNPs, located at 41, 314, 683, and 970 bp position were significantly associated with PL (Fig. 3c). Three of these SNPs (314, 683, and 970 bp) were in complete LD and explained 3.9–8.9% of phenotypic variation in both years. The other SNP (41 bp) explained 3.8–5.3% of phenotypic variation in both years (Table 2). One SNP in *FON1*, at position 1062, was significantly associated with PL (Fig. 3d) and explained 3.6–5.6% of phenotypic variation in both field tests (Table 2). The G allele at this position was exclusively existed in *FON1–4* haplotype. In *HD1*, significant associations were detected between 14 polymorphic sites and phenotypic value of PB and SB (Fig. 3e). Of these, ten polymorphic sites in complete LD (248, 249, 440, 466, 469, 487, 512, 661, 1062, and 1324 bp) explained 7–8.9% of phenotypic variation in PB in both years. An INDEL located at 1216 bp position was significantly associated with PB and SB, explaining 5.7–6.8% of variation in PB and 7.8–18.5% of variation in SB in both field tests (Table 2). Two SNPs in *SP1*, located at 922 and 983 bp position were significantly associated with SB and were in complete LD (Fig. 3f). These polymorphic sites, found exclusively in the *SP1–5* haplotype, explained 12.6–17% of variation in SB in both field tests (Table 2).

**Table 2:** Sequence variants associated with three panicle-related traits in two field tests

2013							2014					
Site	PL		PB		SB		PL		PB		SB	
	*p*	*r* ^*2*^	*p*	*r* ^*2*^	*p*	*r* ^*2*^	*p*	*r* ^*2*^	*p*	*r* ^*2*^	*p*	*r* ^*2*^
APO1												
11 sites in LD^a^	–	–	–	–	0.001	0.180	–	–	–	–	0.014	0.109
948					0.003	0.191					0.032	0.123
APO2												
102, 888^b^	–	–	–	–	0.007	0.123	–	–	–	–	0.012	0.112
DEP1												
41	0.003	0.053	–	–	–	–	0.035	0.038	–	–	–	–
314^c^	0.011	0.039	–	–	–	–	0.005	0.064	–	–	–	–
683, 970^c^	0.006	0.060	–	–	–	–	0.004	0.089	–	–	–	–
FON1												
1062	0.045	0.036	–	–	–	–	0.024	0.056	–	–	–	–
HD1												
10 sites in LD^d^	–	–	0.001	0.089	–	–	–	–	0.011	0.070	–	–
646	–	–	0.006	0.064	–	–	–	–	0.012	0.067	–	–
652	–	–	0.015	0.052	–	–	–	–	0.010	0.071	–	–
660	–	–	0.005	0.088	–	–	–	–	0.012	0.093	–	–
1216	–	–	0.005	0.068	0.001	0.185	–	–	0.023	0.057	0.039	0.078
SP1												
922, 983^e^	–	–	–	–	0.001	0.170	–	–	–	–	0.008	0.126

a, 11 sites in complete LD: 40, 57, 537, 546, 627, 765, 795, 850, 951, 1005, 1188; d, 10 sites in complete LD: 248, 249, 440, 466, 469, 487, 512, 661, 1062, 1324

^a^, ^b^, ^c^, ^d^, and ^e^ were in complete LD

### Combination of favorable alleles

**Fig. 4:**
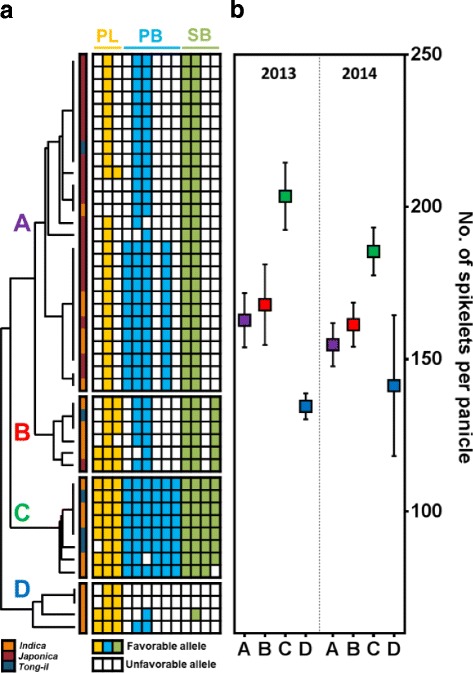
Unweighted Pair Group Method with Arithmetic Mean (UPGMA) cluster analysis. **a** Allele combinations associated with panicle-related traits were divided into four clusters. Colors indicate favorable alleles for panicle-related traits: yellow, PL; sky blue, PB; yellow-green, SB. **b** Comparisons of average SPP values between clusters for 2 years (mean ± SEM)

Based on the estimates of the allelic effect on phenotypic variation for panicle-related traits, each of variants were classified as favorable or unfavorable allele (Table 3). UPGMA cluster analysis divided allele combinations into four clusters according to polymorphic sites with consistent effects on phenotypic variation in both years (Fig. 4a). Cluster A, 74% of which were *japonica*, carried an average of 5.7 favorable alleles. Cluster B was represented by *indica* varieties (66.7%) and had seven favorable alleles on average. Cluster C contained four *indica* and three *tong-il* varieties and had the largest number of favorable alleles (12 alleles on average). The lowest average number of favorable alleles (4.5) was found in Cluster D. The average SPP value for each cluster was proportional to the number of favorable alleles, and SPP for Cluster C was higher than for other clusters (Fig. 4b).

**Table 3:** Estimates of the allelic effect for phenotypic variation

Position (Position (bp)	Allele	2013			2014		
		PL	PB	SB	PL	PB	SB
APO1							
40^a^	A	–	–	14.4	–	–	9.9
	G	–	–	0.0	–	–	0.0
948	C	–	–	8.9	–	–	15.5
	DEL	–	–	14.6	–	–	9.8
	A	–	–	0.0	–	–	0.0
APO2							
102^b^	G	–	–	13.2	–	–	11.1
	A	–	–	0.0	–	–	0.0
DEP1							
41	A	5.7	–	–	4.3	–	–
	G	0.0	–	–	0.0	–	–
314^c^	A	−7.4	–	–	−8.5	–	–
	G	0.0	–	–	0.0	–	–
683^c^	T	4.2	–	–	4.1	–	–
	A	11.5	–	–	12.5	–	–
	DEL	0.0	–	–	0.0	–	–
FON1							
1062	C	−1.3	–	–	−1.2	–	–
	G	0.0	–	–	0.0	–	–
HD1							
247^d^	G	–	2.1	–	–	1.9	–
	A	–	0.0	–	–	0.0	–
646	G	–	1.9	–	–	2.0	–
	DEL	–	0.0		–	0.0	–
652	G	–	1.9	–	–	2.3	–
	DEL	–	0.0	–	–	0.0	–
660	C	–	1.2	–	–	1.7	–
	G	–	2.7	–		2.9	–
	DEL		0.0	–	–	0.0	–
1216	A	–	−1.8	−11.9	–	−1.7	−6.8
	DEL		0.0	0.0	–	0.0	0.0
SP1							
922^e^	G	–	–	−10.5	–	–	−8.0
	A	–	–	0.0	–	–	0.0

^a^, 11 sites in complete LD: 40, 57, 537, 546, 627, 765, 795, 850, 951, 1005, 1188; ^b^, complete LD with 888; ^c^, complete LD with 970; ^d^, 10 sites in complete LD: 248, 249, 440, 466, 469, 487, 512, 661, 1062, 1324; ^e^, complete LD with 983

## Discussion

Characterizing the genetic diversity underlying agronomic traits provides evolutionary context and uncovers beneficial sequence variants that can be used to improve crop varieties. Understanding the relation between specific alleles and phenotypic variation facilitates the development of effective markers for use in rice breeding programs [12]. In this study, nucleotide and haplotype diversity of ten genes involved in panicle development were analyzed. Different patterns of sequence variation were observed in the coding regions of these genes. *APO2* exhibited high levels of nucleotide diversity and relatively low haplotype diversity, as characterized by high LD between polymorphic sites (71.8% of pairs of polymorphic sites) (Fig. 3b). By contrast, numerous rare polymorphisms in *DEP1*, *FON1*, and *GN1a* (Table 1) led to the construction of several rare haplotypes and few common haplotypes, resulting in low nucleotide diversity and high haplotype diversity for these genes. Neutrality tests were performed for eight of the ten genes (all except *LAX1* and *MOC1*). Deviation from neutrality was represented using Tajima’s *D* statistic. Significantly positive estimates of Tajima’s *D* were detected only for *APO2*, *FON1*, and *HD1*. No significant deviation from neutrality was detected in the other genes, indicating that population subdivision events or balancing selection occurred at those loci during evolutionary and breeding processes (Table 1). Of the genes examined, the least polymorphism was detected in the *LAX1* coding region, with only a single SNP (Table 1). The high degree of sequence conservation in *LAX1* was detected across the *indica*, *japonica*, and *tong-il* varieties and was thus suggestive of strong selection at the *LAX1* locus during rice breeding.

The *SCM2* allele of *APO1,* which was isolated from ‘Habataki’, a high-yielding Japanese *indica* variety, increased SPP. ‘Koshihikari’ near isogenic lines (NILs) introgressed with *SCM2* showed significantly increased spikelet numbers and grain yield, indicating that the allele could improve yield in *japonica* varieties [33]. In this study, the *APO1–3* haplotype, which was identical to *SCM2*, was detected only in *indica* and *tong-il* accessions. *Tong-il* type varieties, which were derived from crosses between *indica* and *japonica* varieties developed in Korea, exhibited longer panicles, thicker neck nodes, and higher numbers of total rachis branches than *japonica* varieties [34]. Our results thus suggested that *APO1–3* was introgressed from *indica* to *tong-il* type and was artificially selected as a beneficial haplotype during cross breeding between subspecies. In addition, similar patterns were detected in *APO2–3*, *FON1–4*, and *SP1–5*. With the exception of ‘Hanmaeum’, which exhibited the lowest SPP among the *tong-il* type varieties, all *tong-il* type varieties carried the same *indica-*derived haplotypes (Fig. 2). This result implies that these haplotypes, namely, *APO1–3*, *APO2–3*, *FON1–4*, and *SP1–5*, are preferred in *tong-il* type varieties and could contribute to the development of high-yielding varieties in crosses between *indica* and *japonica* rice cultivars.

Candidate gene association analysis is an effective way to identify favorable alleles for target traits. This approach has been extensively applied to discovering sequence variants associated with many rice traits, such as plant height, flowering time, spikelet number [14], disease resistance [35], and starch characteristics [36, 37]. Comprising association panel with a wide range of phenotypic variations is important to have enough statistical power to detect associated variants. Despite relatively small sample size, several previous studies successfully identified the variants associated with grain quality [38], eating and cooking qualities [39], salt stress resistance [40, 41], and high temperature stress tolerance [42] by using association panel displaying a wide range of phenotypic variations.

In this study, using 45 selected from 205 rice varieties which fully representing phenotypic variation for panicle size, associations between panicle-related traits (PL, PB, and SB) and allelic variants were assessed to identify beneficial sequence polymorphisms with the potential to improve SPP. To reduce the risk of false positive association, we evaluated population structure with admixture model. Estimated Q matrix was used as covariate for the GLM association analysis. In addition, we only used common sequence variants to detect true association, removing minor sequence variants less than MAF 5%.

Five, fourteen, and sixteen polymorphic sites in coding region were significantly associated with PL, PB, and SB, respectively. Some of these sites, however, could not directly affect phenotype. These polymorphic sites can be interpreted as a result of strong LD with trait-related sequence variants [43]. For example, 11 polymorphic sites in complete LD in the *APO1* coding region were significantly associated with SB. Of these, only two non-synonymous SNPs located at 40 bp and 765 bp, and two INDELs located at 850 bp and 1188 bp position led to amino acid sequence changes and were thus likely to affect SB phenotypic variation.

In the *HD1* coding region, four polymorphic sites, including two large INDEL regions of 129 bp and 33 bp, and a small 4 bp INDEL causing a frameshift, were significantly associated with PB. The 4 bp INDEL was also associated with SB under natural long-day conditions in the experimental field in Suwon, South Korea (37°N latitude). These results were consistent with previous reports revealing that large INDELs and small frameshift-inducing INDELs led to partial or complete loss of *HD1* function [15, 44]. Nine non-synonymous SNPs in complete LD in *HD1* were also significantly associated with PB. These SNPs could be attributed to change of *HD1* protein function as they substituted several amino acid sequence. However, it was unclear whether phenotypic variation was due to altered protein function from amino acid substitutions or to strong LD (*r*^2^ = 0.91) with an 2 bp INDEL inducing loss of function, located at 1000 bp (Fig. 3e). Further studies are required to establish the role of the nine non-synonymous SNPs in determining phenotypic variation in PB.

UPGMA cluster analysis classified favorable allele combinations into four clusters (Fig. 4a). Varieties containing the highest number of favorable alleles were located in Cluster C. Varieties in this cluster also exhibited higher SPP values for two field test than varieties from the other three clusters (Fig. 4b). This result suggests that stacking and combining favorable alleles for three panicle-related traits can contribute to increases in SPP even though each individual allele has a relatively minor effect on phenotype (Table 3). Accordingly, the favorable alleles identified in this study can each be used as resource of functional markers in molecular breeding programs for improving SPP. Furthermore, combining favorable alleles from multiple genes has the potential to produce greater breeding improvements than using single favorable alleles alone.

## Conclusions

A total of 142 polymorphic sites, which constructed 58 haplotypes, were detected in coding regions of several genes involved in panicle development. Thirty-five sequence variants of six genes that were significantly associated with panicle-related traits influenced SPP. Although each of the associated alleles explained relatively small amounts of phenotypic variation, a group of cultivars carrying higher numbers of favorable alleles exhibited higher SPP values on average than groups with fewer favorable alleles. This result implies that SPP could be increased by stacking favorable alleles for panicle-related traits. The favorable alleles identified in this study can therefore be used as resource for functional markers, and stacking favorable alleles could contribute to SPP improvement in rice breeding programs.

## Additional files

Additional file 1: Distributions of the panicle size of 205 varieties and 45 selected varieties. (PDF 296 kb)

Additional file 2: General information for total accessions used in this study. (XLSX 16 kb)

Additional file 3: Primers used in this study. (PDF 22 kb)

Additional file 4: FASTA format files containing coding sequences for 9 genes. (ZIP 19 kb)

Additional file 5: Results of sequence analysis of *MOC1* and *LAX1* coding region. (PDF 420 kb)

Additional file 6: Summary of statistics for four traits of 45 varieties. (XLSX 10 kb)

Additional file 7: Haplotypes of four genes. (PDF 197 kb)

Additional file 8: Plots of Evanno’s delta K (∆K). (PDF 115 kb)

## Abbreviations

GLM: General Linear Model
LD: Linkage disequilibrium
MAF: Minor allele frequency
NIL: Near isogenic line
PB: The number of primary branches per panicle
PL: Panicle length
SB: The number of secondary branches per panicle
SPP: The number of spikelets per panicle
UPGMA: Unweighted Pair Group Method with Arithmetic Mean
