# Supplementary material for: Association between sequence variants in panicle development genes and the number of spikelets per panicle in rice
Source: BMC Genet. 2018 Jan 15;19:5. doi: 10.1186/s12863-017-0591-6 (PMC5769279; doi:10.1186/s12863-017-0591-6)
Supplement: Supplementary file 1 — Distributions of the panicle size of 205 varieties and 45 selected varieties. (PDF 296 kb) [file 12863_2017_591_MOESM1_ESM.pdf]

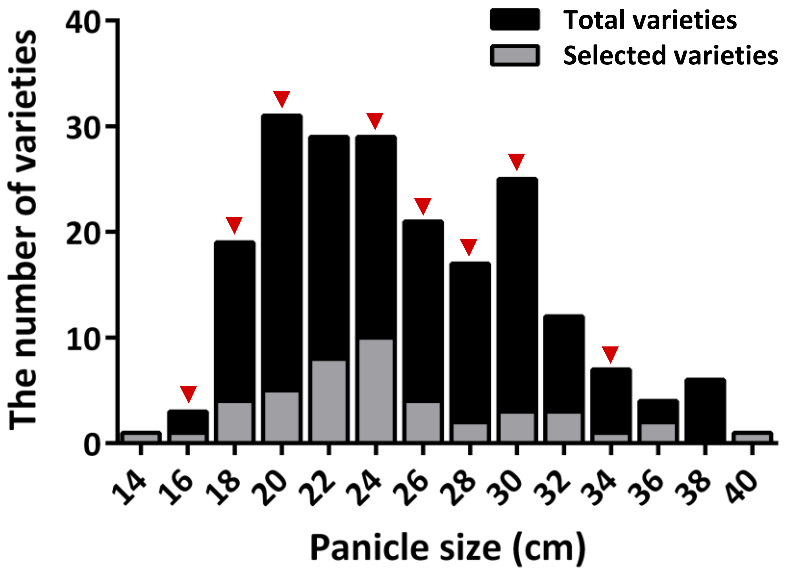

Additional file 1 Distributions of the panicle size of 205 varieties and 45 selected varieties. Blac k bar and grey bar indicate total varieties and selected varieties, respectively. Eight representative varieties were sequenced to find sequence variants in coding region of *MOC1* gene (red triangle). Representative varieties are DL 1, Sindongjin, Deshi Boro, Cibogo, Hangangchal 1, Kashmir Basmati, Calotoc, and Kiwisan from the left
