# Supplementary material for: Association between sequence variants in panicle development genes and the number of spikelets per panicle in rice
Source: BMC Genet. 2018 Jan 15;19:5. doi: 10.1186/s12863-017-0591-6 (PMC5769279; doi:10.1186/s12863-017-0591-6)
Supplement: Supplementary file 3 — Primers used in this study. (PDF 22 kb) [file 12863_2017_591_MOESM3_ESM.pdf]

Additional file 3 Primers used in this study

Gene	RAP-DB ID	Primer name	Primer sequence		Product size (bp)
			Forward (5'-3')	Reverse (5'-3')	
APO1	Os06g0665400	cds_apo1	TCACATGAATTAATCCCTTGGCATC	ATATGATCCCCATTGCTGAAACAAC	1680
APO2	Os04g0598300	cds_apo2_1	GCAACGAGCTATGGAGATTTGTC	TCGTCTTACGTCTGCATGCTTAT	1317
		cds_apo2_2	GGTGATTGGATGGCTTTGCATC	GGCCATGAGGATATGTCACGAT	991
Ghd8	Os08g0174500	cds_ghd8_1	TAGTAGCGTCCTTATGTTTGCTTTG	GGATCACAACCGAACTCCTACAG	1263
		cds_ghd8_2	CGAAACTGCAAACCATGTGTAGGAC	CAACGCCAAGTTACGTGCCAG	501
Hd1	Os06g0275000	cds_hd1_1	GAGGTAGAGGAACAGGAGAAGACG	TTTAATCAGCCTAAAGATCGCAGC	1214
		cds_hd1_2	TACACAGCAATCACCACACGAAAG	CTCATCACTGCTCTTTGCTTACTTC	854
Gn1a	Os01g0197700	cds_gn1a_1	CACCACAGCTCTACTGTCTATCTAG	CTTCTCCTTCGAGCACGTCAC	947
		cds_gn1a_2	GTGTCGTGGACAGACTACCTC	TACTTCCAGTTAGGTGTGTGATAGC	1277
		cds_gn1a_3	CTGTCCATCCACGCTGCTAG	GCTTGACTAGAGCATTGTCCATTG	1326
FON1	Os06g0717200	cds_fon1_1	CATTGACCACGCCAACCTAAC	CGACGAAGTCCGGTATGCTG	1176
		cds_fon1_2	TACATCGGATACTACAACCAGTACG	CTCGTTGAACACCAAGAACTGC	1146
		cds_fon1_2-3	GAGCATCACGTCGCTCAAGATAC	CAGGATAAACTTGTTGGGTCTTGG	1126
		cds_fon1_3	AAATGTGCAGCTCTAATGGCTGTAG	AGCAGTAGTAATCCGCCTGTTATTG	645
SP1	Os11g0235200	cds_sp1_1	AAGCACAAAGCAAGCACTATCAC	CAAGGAGCCAAGAAATGCAGTC	777
		cds_sp1_2	TTTCGAGCAGGTTCATTGCATC	GTACGACGATTGTATTGTACGGC	655
		cds_sp1_3	AATTACCACTGTCTGCTGGGAGG	ATGGAGAAGGTGACGGCGAAGAG	979
		cds_sp1_4	GTGAAGGTGCTACTGTGCG	GCATCCTTGCAGAGTCAAAC	1082
DEP1	Os09g0441900	cds_dep1_1	CTCTCCATCTCCGCTGCTATTATTG	GTACACTTCACAGCATCCTACTTG	697
		cds_dep1_2	TTCGTACTGGTCAATTACTCCAGG	AGAGCATAAGACATTAGATAAGGACAG	553
		cds_dep1_3	CCGATTCTTTCCATGCGATGTG	AATTGGTAGAGCACGAGCCAC	1486
		cds_dep1_4	ACTTGCACCCTTCCAAGCTGTAGC	TCATCATTTCTTTACAGGATAAACACC	719
LAX1	Os01g0831000	utr_lax1	AAGTTATGAGCACGAGAATTACAG	CGTGAATACGTGAGCTACCATC	1649
MOC1	Os06g0610350	cds_moc1	CATGATGACGTAAACGAATCTCGG	ACGAATAAAATTGCACATATAGAGGG	505
		asp_moc1_g	AAAACATGAGATGGTTGGACAG	TGCATGGAAACCTTTTTAACG	200
		asp_moc1_c	AAAACATGAGATGGTTGGACAC		
		common_moc1	CGTCCATTTCTCGATCCAAT		498
