# Supplementary material for: Association between sequence variants in panicle development genes and the number of spikelets per panicle in rice
Source: BMC Genet. 2018 Jan 15;19:5. doi: 10.1186/s12863-017-0591-6 (PMC5769279; doi:10.1186/s12863-017-0591-6)
Supplement: Supplementary file 5 — Results of sequence analysis of MOC1 and LAX1 coding region. (PDF 420 kb) [file 12863_2017_591_MOESM5_ESM.pdf]

A

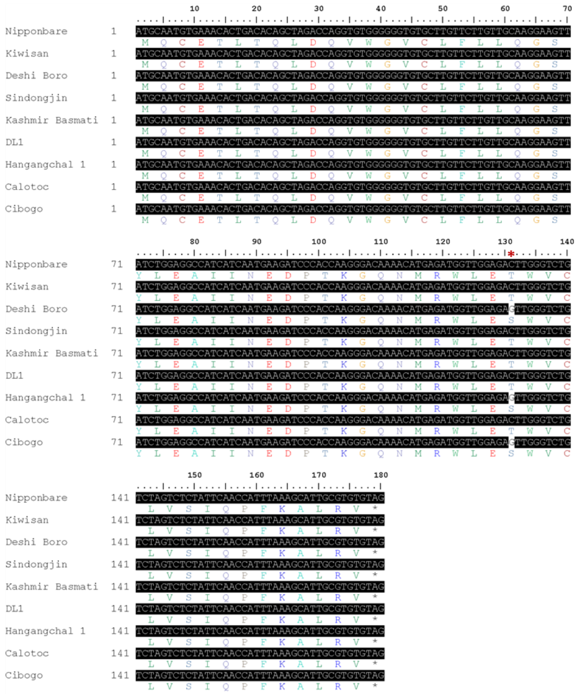

B

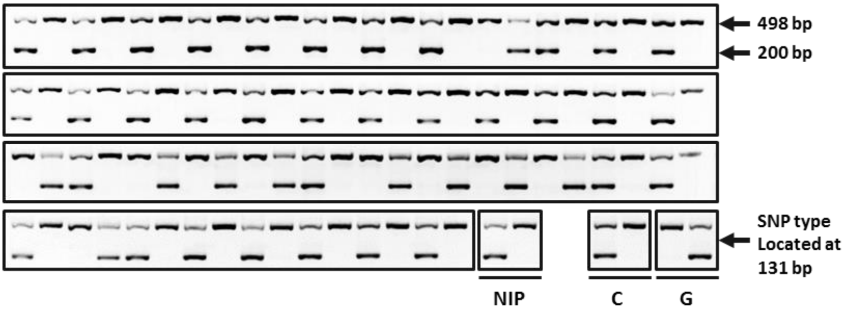

C

Region		EXON 1
Position		349
NIP		T
Type 1	*LAX1-T*	T
Type 2	*LAX1-G*	G

Additional file 5 Results of sequence analysis of *MOC1* and *LAX1* coding region. (A) Multiple sequence alignment of *MOC1* coding region. Red asterisk denotes position of non-synonymous SNP which leads to change of amino acid sequence. Results of genotyping by allele-specific markers for (B) *MOC1* and sequencing for (C) *LAX1*
