# Supplementary material for: Association between sequence variants in panicle development genes and the number of spikelets per panicle in rice
Source: BMC Genet. 2018 Jan 15;19:5. doi: 10.1186/s12863-017-0591-6 (PMC5769279; doi:10.1186/s12863-017-0591-6)
Supplement: Supplementary file 7 — Haplotypes of four genes. (PDF 197 kb) [file 12863_2017_591_MOESM7_ESM.pdf]

A

Region		EXON 1	EXON 5																
Position		41	314	318	587	589	590	591	592	593	594	597	598	599	611	683	782	833	970
Nip		A	A	T	TT	T	C	A	T	G	C	C	A	T	G	T	G	CG	T
Type 1	*DEP1-3*	A	A	T	TT	T	C	A	T	G	C	C	A	T	G	T	G	CG	T
Type 2	*DEP1-1*	A	A	T	AG	A	T	C	C	T	T	T	T	625 del					
Type 3	*DEP1-4*	G	A	T	TT	T	C	A	T	G	C	C	A	T	G	T	G	CG	T
Type 4	*DEP1-6*	A	G	T	TT	T	C	A	T	G	C	C	A	T	A	A	G	GG	A
Type 5	*DEP1-9*	A	G	T	TT	T	C	A	T	G	C	C	A	T	A	A	G	CG	A
Type 6	*DEP1-5*	A	G	T	TT	T	C	A	T	G	C	C	A	T	G	A	G	CC	A
	DEP1-2	A	G	C	TT	T	C	A	T	G	C	C	A	T	G	A	G	CG	A
Type 7	*DEP1-8*	A	G	C	TT	T	C	A	T	G	C	C	A	T	G	A	A	CG	A
Type 8	*DEP1-7*	A	G	C	TT	T	C	A	T	G	C	C	A	T	G	A	G	GG	A

B

Region		EXON 1					EXON 2		EXON 3		EXON 4	
Position		161	235	319	353	567	741	828	1247	1346	1466	1613
Nip		G	-	G	A	C	C	C	-	C	G	T
Type 1	*GN1a-3*	G	-	G	A	C	C	C	-	C	G	T
Type 2	*GN1a-7*	C	12 del	G	G	C	C	C	-	C	G	G
	GN1a-5	C	12 del	G	G	C	A	A	-	C	G	G
	GN1a-2	C	12 del	G	G	C	C	C	-	C	A	G
Type 3	*GN1a-4*	C	6 ins	G	G	C	C	C	-	C	G	G
Type 4	*GN1a-6*	C	6 ins	T	G	C	C	C	-	C	G	G
Type 5	*GN1a-8*	C	6 ins	T	G	C	C	C	2 ins	C	G	G
Type 6	*GN1a-1*	C	6 ins	G	G	T	C	C	-	1 del	A	G

C

Region		EXON 1																			
Position		26	56	74	96	132	177	222	256	306	323	461	536	569	651	659	674	819	828	878	893
Nip		A	T	A	T	C	G	G	T	A	A	A	GCG	A	C	A	A	-	C	TT	A
Type 1	*GHD8-1*	A	T	A	T	C	G	G	T	A	A	A	GCG	A	C	A	A	-	C	TT	A
	GHD8-4	A	T	A	T	C	G	T	T	A	A	A	GCG	A	C	A	A	-	C	TT	A
Type 2	*GHD8-9*	A	T	A	T	C	C	G	T	A	A	A	GCG	A	C	A	A	-	C	TT	A
Type 3	*GHD8-5*	A	C	C	G	C	G	G	G	C	A	C	2 GCG	C	T	C	A	-	9 del	TT	A
Type 4	*GHD8-7*	A	C	C	G	C	G	G	G	C	A	C	2 GCG	C	T	C	A	-	C	TT	G
Type 5	*GHD8-2*	A	C	C	G	C	G	G	G	C	A	C	2 GCG	C	T	C	A	-	9 del	TT	G
Type 6	*GHD8-3*	A	C	C	G	C	G	G	G	C	1 del	C	2 GCG	C	T	C	A	-	9 del	TT	G
Type 7	*GHD8-8*	A	C	C	G	C	G	G	G	C	A	C	6 del	C	T	C	A	-	C	TT	G
Type 8	*GHD8-6*	T	C	C	G	T	G	G	G	C	A	C	6 del	C	C	C	1 del	6 ins	C	GA	G

D

Region		EXON 1														EXON 2				
Position		71	248	316	334	440	466	469	487	512	517	646	660	661	779	1000	1062	1170	1216	1324
Nip		-	GC	C	-	A	C	C	G	A	-	-	C	-	G	TT	A	-	-	G
Type 1	*HD1-1*	-	GC	C	-	A	C	C	G	A	-	-	C	-	G	TT	A	-	-	G
Type 2	*HD1-3*	-	GC	T	36 ins	A	C	C	G	A	-	-	C	-	G	TT	A	-	-	G
Type 3	*HD1-4*	-	GC	C	36 ins	A	C	C	G	A	-	-	C	-	G	TT	A	-	-	G
Type 4	*HD1-8*	-	GC	C	36 ins	A	C	C	G	A	-	-	G	-	A	TT	A	-	4 del	G
Type 5	*HD1-2*	-	AA	C	36 ins	G	A	A	A	G	-	33 del	-	-	A	2 del	C	-	-	A
Type 6	*HD1-5*	3 del	AA	C	36 ins	G	A	A	A	G	129 ins	-	C	6 del	A	2 del	C	-	-	A
Type 7	*HD1-6*	-	AA	C	36 ins	G	A	A	A	G	129 ins	6 del	C	6 del	A	2 del	C	-	-	A
Type 8	*HD1-7*	-	AA	C	36 ins	G	A	A	A	G	129 ins	-	C	6 del	A	2 del	C	-	-	A
Type 9	*HD1-9*	-	AA	C	36 ins	G	A	A	A	G	-	33 del	-	-	A	TT	C	3 del	-	A

Additional file 7 Haplotypes of four genes. (A) *DEP1* (B) *GN1a* (C) *GHD8* (D) *HD1.* Nip, nipponbare; Type, candidate protein type; ins, insertion; del, deletion. Light blue boxes denote position of non-synonymous polymorphism. Orange box means mutational event causing frame shift and premature stop
