# Supplementary material for: Association between sequence variants in panicle development genes and the number of spikelets per panicle in rice
Source: BMC Genet. 2018 Jan 15;19:5. doi: 10.1186/s12863-017-0591-6 (PMC5769279; doi:10.1186/s12863-017-0591-6)
Supplement: Supplementary file 8 — Plots of Evanno’s delta K (∆K). (PDF 115 kb) [file 12863_2017_591_MOESM8_ESM.pdf]

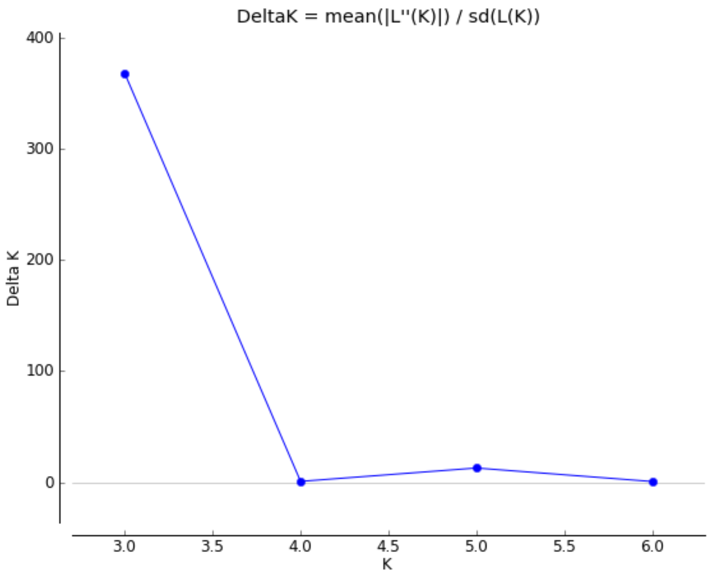

Additional file 8 Plots of Evanno's delta K (ΔK). The most probable structure number was calculated by Evanno’s method
